# Asymmetric Lipid Membranes: Towards More Realistic Model Systems

**DOI:** 10.3390/membranes5020180

**Published:** 2015-05-06

**Authors:** Drew Marquardt, Barbara Geier, Georg Pabst

**Affiliations:** 1 Institute of Molecular Biosciences, Biophysics Division, University of Graz, NAWI Graz, Humboldtstr 50/III, Graz, 8010, Austria; E-Mail: barbara.geier@uni-graz.at; 2 BioTechMed-Graz, Graz, 8010, Austria

**Keywords:** asymmetry, vesicles, model membranes, phospholipids

## Abstract

Despite the ubiquity of transbilayer asymmetry in natural cell membranes, the vast majority of existing research has utilized chemically well-defined symmetric liposomes, where the inner and outer bilayer leaflets have the same composition. Here, we review various aspects of asymmetry in nature and in model systems in anticipation for the next phase of model membrane studies.

## 1. Asymmetry in Natural Membranes: A Brief Introduction

**Figure 1:**
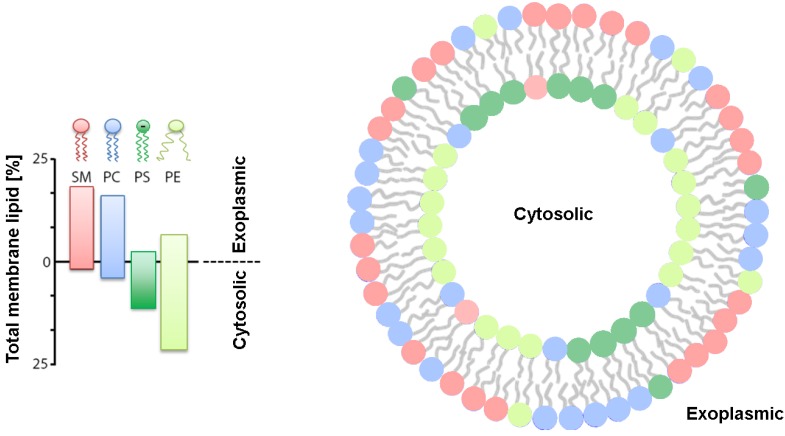
Proposed distribution of phospholipids in human red blood cells put forward by Verkleij *et al.* [4].

Arguably the most notable year in the study of biological membranes was 1972. Not only the archetypal fluid mosaic model of Singer and Nicolson [1] was published in 1972, but Mark Bretscher provided the first report of partial lipid asymmetry in membranes [2,3]. Remarkably, only a year later, quantitative analysis of the asymmetric lipid distribution in various cell types, including human erythrocytes, was determined (Figure 1) [4], piloting membrane research already in its early days and, in particular, membrane biophysics toward deciphering the role of membrane asymmetry.

Like all eukaryotic cells, mammalian plasma membranes (PM) actively sequester nearly all of their sphingomyelin, (SM) and phosphatidylcholine (PC) within the outer monolayer of the membrane. The inner monolayer was determined to consist of phosphatidylethanolamine (PE) and the negatively-charged lipids phosphatidylserine (PS) and phosphatidylinositol (PI) [4,5]. Cholesterol (Chol) is found in both membrane leaflets, but apparently enriched within the inner leaflet [6]. Asymmetry is also observed in bacterial membranes, although it is more difficult to quantify. Nevertheless, it has been reported that PI and PE are preferentially located in the inner leaflet, phosphatidylglycerol (PG) in the outer leaflet, while cardiolipin (CL) is distributed over both leaflets in plasma membranes in Gram-positive bacteria [7,8]. Membrane asymmetry is known to affect various bilayer properties, including membrane potential, surface charge, permeability, shape, as well as stability [5,9,10]. Loss of asymmetry has physiological consequences. For example, PS exposure occurs in mammalian cells during apoptosis and is an important signal for their disposal by macrophages (see, e.g., Fadok and Henson [11]). PS externalization has also been linked to blood coagulation and erythrocyte adhesion (see, e.g., Lentz or Wautier *et al.* [12,13]) and myotube formation [14] and has been reported recently for cancer cells [15].

An asymmetric membrane is a system not at equilibrium, which would prefer a symmetric distribution of membrane lipids. Thus, maintaining membrane asymmetry in live cells is an active process (requiring ATP) carried out by proteins, known as flipases or floppases [16,17]. In addition to transmembrane asymmetry, lateral inhomogeneities in membranes, and, in particular, the formation of functional platforms (domains), such as membrane rafts, have attracted significant interest and research efforts in the past few decades [18]. Membrane rafts are thought to enable the assembly of signaling proteins or transbilayer transport and are enriched in SM and Chol [19]; similarly, recent experiments suggested significant influences originating from the cytoskeleton [20]. Interestingly, several laboratories have reported that lipid-only membranes with a symmetric distribution of outer leaflet lipids (SM, PC) and Chol readily separate into coexisting liquid-disordered (L${}_{d}$) and liquid-ordered (L${}_{o}$) phases over a wide range of compositions and temperatures (for a review, see, e.g., [21]). Moreover, L${}_{o}$ domains were found to be enriched in high-melting lipids, such as SM and Chol [22], thus encouraging their application as simple models to study the properties of membrane rafts. On the contrary, inner leaflet lipid mixtures (e.g., PE, PS and Chol) were found to exhibit complete miscibility, *i.e.*, PE/PS/Chol mixtures form a uniform L${}_{d}$ phase in symmetric bilayers [23]. However, membrane domain formation in the outer leaflet somehow influences the organization of the inner leaflet-associated proteins during signal transduction [24,25]. Understanding the origin of this coupling mechanism is a major challenge in understanding the role of rafts in membrane function. Moreover, these observations suggest an ability of inner leaflet components to sense and respond to the physical state of the outer leaflet components, implying the existence of interleaflet communication. Whether such communication manifests itself in coupling of domain formation across the bilayer or induces other characteristic structural and dynamic changes in the lipids of the two leaflets remains unclear despite research efforts in this direction [26,27,28,29].

Lipid-only model membranes offer unique insight into such interactions from well-defined systems. However, with a few exemptions detailed below, lipids, including complex lipid mixtures, self-assemble into symmetric bilayers, and asymmetry is difficult to establish experimentally without being able to resort to flipases or floppases. Thus, the majority of lipid-only studies have been performed using symmetric lipid membranes. Only recently has there been a new and strong impetus toward studying asymmetric membranes brought about by new and easy to follow protocols. At this dawn of a new era of membrane biophysics, we highlight the progress made and early insights achieved from such model systems, including a brief account of membrane asymmetry originating either from lipid properties or external constraints.

## 2. Asymmetry in Model Membranes

### 2.1 Geometric Asymmetry

**Figure 2:**
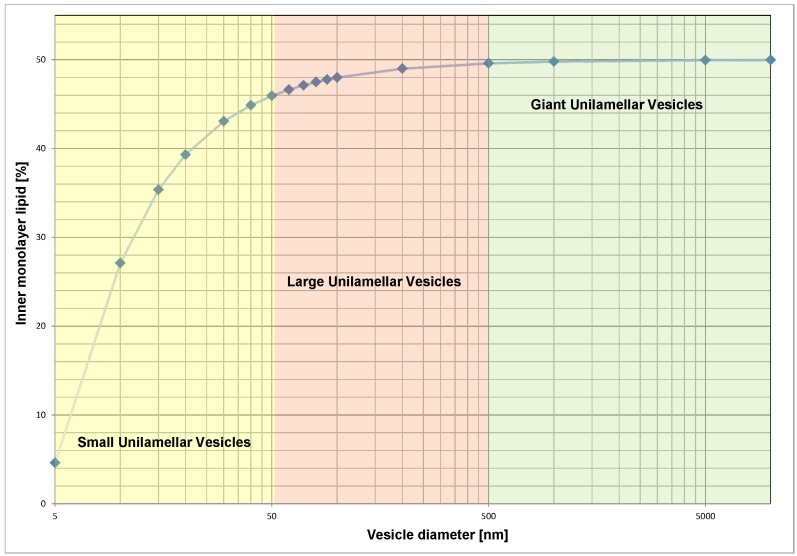
Asymmetric lipid distribution due to vesicle size. Data were generated using the area per lipid and bilayer thickness of 1-palmitoyl-2-oleoyl-sn-glycero-3-phosphocholine (POPC) as determined by Kučerka and co-workers [34].

The most common source of asymmetry, and often overlooked, in model vesicles is the non-equal number of lipid molecules between bilayer leaflets as a result of vesicle size. As the vesicle diameter decreases, the difference in leaflet surface area increases. This difference in surface area is reflected in the number of lipid molecules that exist in each leaflet, which can be calculated based on the structural details of unstressed bilayers (Figure 2). A recent coarse-grained MD simulation demonstrates membrane asymmetry by increasing the lipid density in one leaflet [30]. Lipid number density asymmetry is most easily observed experimentally by means of nuclear magnetic resonance spectroscopy [31,32]. The asymmetry has been shown via the use of a paramagnetic shift reagent, which interacts with the outer monolayer only, creating two separate signals (*i.e.*, the inner and outer leaflet signals separate). In the special case of small unilamellar vesicles (SUV), <50 nm, the asymmetric distribution can be qualitatively observed directly, as the packing of the inner and outer monolayers is different [31,32]. This directly affects the melting transition, which is distinct from unstressed bilayers [33].

A further driving force for membrane asymmetry results from lipid intrinsic curvature leading to lateral and transverse lipid separation [35]. This effect may be also coupled to vesicle size, but does in general not depend on it, as it originates from an intrinsic lipid property. In the original postulation by Chapman, Willaims and Ladbrooke, Equation (1), the angle between the hydrocarbon chain axes and the phospholipid/water interface (τ) is described by the lateral space occupied by the fatty acid chains ($S_{o}$), lipid molecular weight ($M_{W}$), the thickness of lipid layer (*t*)and the lipid density (ρ), where $N_{A}$ is Avogadro's number.

(1) $$\text{τ}=sin^{-1}\left(\frac{tS_{o}\text{ρ}N_{A}}{M_{W}}\right)$$

**Figure 3:**
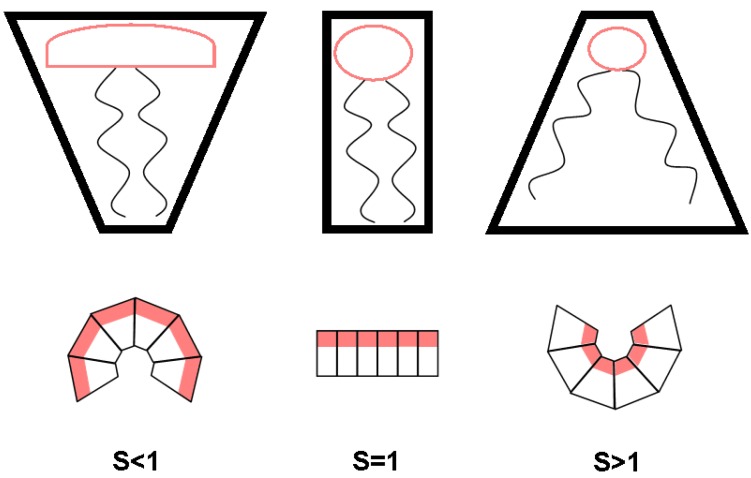
Cartoon illustration of differently-shaped lipids and the associated curvature.

A more familiar form of Equation (1) describes the shape parameter *S*:

(2) $$S=\frac{V}{l_{c}a_{o}}$$

where $a_{o}$ is the optimum area per molecule at the lipid/water interface, *V* is the volume per molecule and $l_{c}$ is the length of the fully-extended acyl chain [2]. Phospholipids with a shape parameter of S<1 adopt a cone-like shape, which would correspond to an area of positive curvature (Figure 3). Shape parameters that are S=1 correspond to a cylindrical shape preference, which would result in domains of neutral curvature. Finally, S>1 corresponds to an inverted cone shape preference leading to negative curvatures.

Although headgroup and tail composition both play a role in determining the shape parameter, a general rule is that PC lipids, as well as SM form regions of neutral or positive curvature, whereas PS and PE form neutral to negative regions of curvature, which explains the predominance of PS and PE on the inner monolayer of the PM [4,5], at least from a plain physical point of view. Quantitative assessment of intrinsic curvature for different phospholipids has been reported recently by Kollmitzer *et al.* [36].

### 2.2. Cholesterol Distribution

Most of the currently available data suggest that cholesterol is asymmetrically distributed in membranes; however, which leaflet Chol resides in is still debated. Several studies on natural plasma membranes, using quenching of dehydroergosterol (a natural fluorescent sterol found in sponge and yeast [37]) suggest that Chol mainly resides in the inner leaflet (see, e.g., [6] for a review). Chol was observed to be distributed asymmetrically in mono-unsaturated PC lipids. Unfortunately, the authors could only speculate that Chol partitions preferentially to the inner monolayer [38], which was based on work by Huang and co-workers demonstrating that Chol concentrates in regions of high curvature [39]. Giang and Schick [40] summarize the corresponding theoretical framework explaining the affinity of Chol for regions of high curvature. The theory suggests that PE, which is known to predominantly exist in the inner leaflet of the PM [4,5] and forms regions of high negative curvature (S>1), draws Chol to the inner leaflet to lower the bending free energy. Interestingly, when applied to biologically-relevant systems, such as human erythrocytes, a nearly symmetric Chol distribution, that is only 50%–60% of the Chol should reside in the inner monolayer of the PM, was observed [40].

Chol sequestered to the inner half of the bilayer is contrary to numerous biophysical studies on lipid-only bilayers. These studies report a tight interaction of Chol with SM [41], which locates almost exclusively to the outer leaflet [42,43,44]. In fact, coupling of Chol and SM is one of the foundations of the raft hypothesis [18]. To relieve this disparity, it has been suggested that Chol might interact equally well with the saturated acyl chains of inner monolayer lipids [45].

Interesting insight comes from a highly-detailed coarse-grain MD simulation [46], studying Chol location and dynamics in a number of asymmetric bilayers of differing leaflet compositions. The simulations demonstrate that Chol adopts an asymmetric distribution upon reaching equilibrium after up to 10 μs (all of the systems were simulated for 12–15 μ*s*) [46]. The equilibrium location of Chol was found to depend not only on the leaflet on which it resides, but also on the composition of the other leaflet, demonstrating that an asymmetric bilayer must be viewed as one entity and not as being composed of two non-communicating leaflets (discussed further in Section 3.2). When reconciling model system data with PM observations, one must keep in mind that the PM is far more complex, and cholesterol asymmetry could be organized differently compared to large unilamellar vesicles (LUVs) and MD simulations.

### 2.3. Charge

Small angle X-ray scattering (SAXS) experiments on liposomes composed of PS lipids have reported that the scattered intensity does not approach zero between the second and third lobe, indicating an asymmetric electron density profile [47,48]. This feature in the scattering intensity could in principle also originate from constraints from vesicle size, as discussed in Section 2.1. However, zwitterionic PC vesicles of the same size, that is LUVs, exhibit a minimum with zero scattered intensity in this regime, revealing that the asymmetry is not imposed by bilayer curvature [47]. Furthermore, the coexistence of an interdigitated and a non-interdigitated gel phase, which was reported for PG lipids by Pabst *et al.* [49], can be ruled out for fluid PS bilayers. Instead, asymmetry must originate from some lipid property. Brzustowicz *et al.* have demonstrated from fitting their SAXS data of 100 nm 1-stearoyl-2-oleoyl-sn-glycero-3-phospho-L-serine (SOPS) LUV that the inner leaflet is more disordered, suggesting that SOPS vesicles are rougher on the inner leaflet compared to the outer [48].

On the other hand, it is well known that the charge state of a lipid depends strongly on the pH. For example, the relative charge goes from +1 at a pH value of one to−2 for a pH value of 13 for PS [50,51]. A different charge state leads to a change in headgroup sizes, which again cause a change of the shape (see Figure 3) [52]. In support of this idea, Hope *et al*. reported that a transbilayer pH gradient can induce an asymmetric lipid distribution between the inner and outer leaflet [53].

## 3. Synthesized Asymmetry

### 3.1. Construction of Asymmetric Vesicles

Aside from the aforementioned special conditions, several techniques have been developed to realize free-floating asymmetric lipid-only membranes [10,28,54,55,56,57,58,59], opening up several ways to study the role of membrane asymmetry.

**Figure 4:**
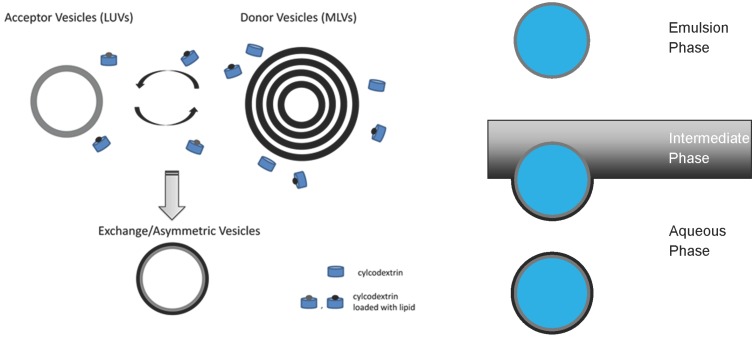
Schematic of asymmetric vesicle construction methods. The left is a schematic of cyclodextrin-mediated lipid exchange of the outer membrane leaflet [10]. The right-hand scheme is the engineering of asymmetric vesicles generated by two independently prepared monolayers [54].

Most promising in this respect appears to be important progress made in the laboratory of Erwin London (Stony Brook, NY, USA) using cyclodextrin-mediated lipid exchange, as depicted in Figure 4 (left) [10,28,57,58,59]. Recently, the protocol has been adapted for the construction of asymmetric supported bilayers [60]. The technique can be applied to construct free-floating asymmetric lipid vesicles with various headgroup and acyl chain compositions containing Chol without the removal of Chol from the model membrane by cyclodextrin [58,59,61]. This has opened a new window to several biophysical studies, including the reconstitution of membrane proteins, as discussed further below.

Weitz and co-workers engineered asymmetric free-floating vesicles by means of two independently prepared lipid monolayers, as shown in Figure 4 (right) [54]. This methodology is similar to the Langmuir–Blodgett/Langmuir–Schaefer techniques for asymmetric supported bilayers [62,63]. Although less “violent” than cyclodextrin-mediated asymmetric vesicle construction, this preparation is not without its drawbacks. Most notable is the possible presence of silicon oil in the final vesicle solution. A drawback that synthetic asymmetric liposomes suffer is time-induced loss of asymmetry. Like real PM, these synthetic asymmetric liposomes are not in a state equilibrium,; thus, the ‘lifetime’ of the asymmetry is limited. To the best of our knowledge, this ‘lifetime’ is limited to the order of hours.

### 3.2. Leaflet Coupling

Some of the aforementioned preparations of asymmetric model membranes were used to investigate, by fluorescent dye partitioning, if domain formation in outer leaflet lipids leads to domain formation in the inner leaflet. This behavior was confirmed from a mixture of DOPC/DPPC/Chol (DOPC, 1,2-dioleoyl-sn-glycero-3-phosphocholine; DPPC, 1,2-dipalmitoyl-sn-glycero-3-phosphocholine) in the outer leaflet and DOPC/DOPE/DOPS(DOPE, 1,2-dioleoyl-sn-glycero-3-phosphoethanolamine; DOPS, 1,2-dioleoyl-sn-glycero-3-phospho-L-serine) in the inner leaflet, but not for brain PC/brain SM/Chol in the outer and DOPC in the inner side [26]. Other fluorescence experiments [27] have shown that when domains are present in asymmetric membranes, each leaflet contains regions of three distinct lipid compositions ($L_{o}^{outside}$/$L_{o}^{inside}$, $L_{o}^{outside}$/$L_{d}^{inside}$, $L_{d}^{outside}$/$L_{d}^{inside}$). Such studies have been limited, however, by the lack of methods to prepare asymmetric lipid bilayers with a wide variety of lipid compositions and a highly controlled lipid distribution in each leaflet.

**Figure 5:**
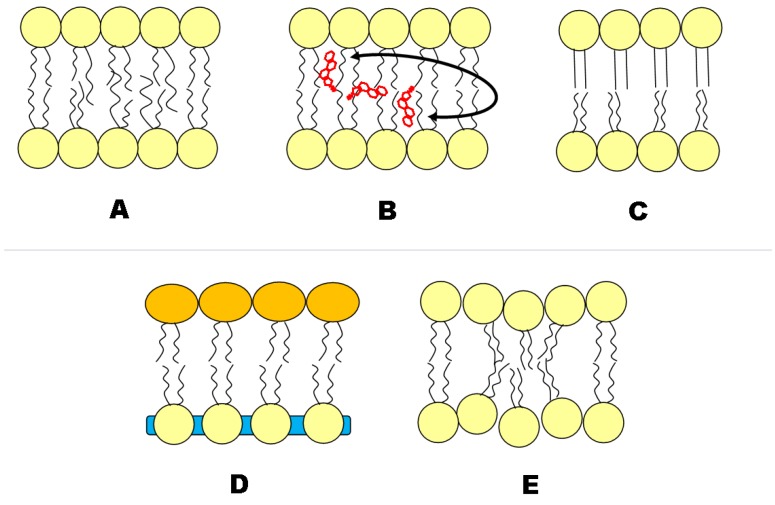
Putative transbilayer coupling mechanisms in asymmetric lipid membranes. (A) Asymmetric lipids containing fatty acid chains of differing length have been observed to interdigitate; (B) Trans-bilayer cholesterol (Chol) movement; (C) the ordered leaflet induces order to the less ordered leaflet; (D) the area per lipid of one leaflet influences the area per lipid of the other, and *vice versa*. Such an area difference can arise from lipid species or charge differences between monolayers; (E) Curvature and/or microemulsion in one leaflet can be transferred to the other.

The effects of asymmetry on the properties of each leaflet have been examined both experimentally and by simulations. Figure 5 surmises presently discussed interleaflet coupling mechanisms. For example, using fluorescence anisotropy measurements, it has been found that SM in the outer leaflet of asymmetric bilayers melts independently of unsaturated PC in the inner leaflet [10,28,59,61]. A coupling between lateral diffusion in the outer and inner leaflet was observed for bilayers composed of lipids with mixed acyl chains (saturated/unsaturated, long/short), suggesting a hydrocarbon chain interdigitation-mediated mechanism [64], in support of theoretical considerations [65].

Other theoretically considered coupling mechanisms involve electrostatic coupling of charged bilayers and Chol flip/flop [65] or van der Waals interactions and composition-curvature coupling [66]. Curvature coupling may result from height-mismatches of L${}_{o}$/L${}_{d}$ domains or long-range tilt correlations in L${}_{o}$ phases, as reported from coarse-grained [67] and all-atom [68] molecular dynamics (MD) simulations. In fact, Polley and co-workers demonstrate that long-range tilt correlations are purely a product of the asymmetric bilayer; tilt correlations were not observed in the symmetric control simulations [68].

An MD study by Bhide *et al.* has shown increased hydrogen bonding (one extra H-bond) in the non-SM leaflet of an asymmetric bilayer. The extra H-bond is most likely due to the ordering effect that the SM leaflet has on the other [69]. The ordering effect of the SM leaflet was also reflected in the order parameter profile for the stearoyl chains in the non-SM monolayer [69]. However, in the study of Bhide, no further interactions between leaflets, such as interdigitation, were observed during the duration of their simulations.

Recently, Shlomovitz and Schick [70] proposed leaflet coupling via the formation of microemulsions in the inner leaflet, which propagate to the outer leaflet. This theory differs from most others, because the driving force of these microemulsions originates from properties of the inner leaflet lipids, not the outer leaflet lipids [70]. It is hypothesized that these microemulsions arise from the coupling of bilayer height fluctuations to lipid number density fluctuations on the inner monolayer [70].

In any case, coupling in asymmetric membranes must involve lateral and transverse correlations. To address transmembrane and intramembrane coupling mechanisms, experimental data of asymmetric membranes with high structural resolution (subnanometer) are needed, but currently lacking.

### 3.3. Flip/Flop

Historically, passive phospholipid flip/flop rates have been challenging to obtain. Researchers strive to address long-standing questions regarding the influence of bilayer structure and composition (including the presence of transmembrane proteins) on flip/flop rates. Much of the flip/flop data are not without significant problems; for example, the frequent use of bulky lipid fluorescence dyes or spin labels can drastically alter the physical properties of the model membrane formed. A primary example of the influence of chemical probes on flip/flop rates was brought forward by Liu and Conboy [71], who determined the flip/flop rate in isotopically asymmetric supported bilayers of DPPC interrogated with sum-frequency vibrational spectroscopy (SFVS). The addition of a common spin-label (*n*,*n*-dimethyl-n-(2’,2’,6’,6’-tetramethyl-4’-piperidyl) (TEMPO)) to DPPC altered the measured flip/flop half-life by an order of magnitude compared to label-free measurements. Table 1 summarizes some membrane monolayer-to-monolayer transitions of phospholipids of various properties (this list is by no means exhaustive).

**Table 1:** Flip/flop half-lives $t_{1/2}$ from various studies with correlation to membrane thickness ($d_{B}$) and temperature (*T*).

Lipid	*t*_1/2_ (min)	*d_B_* (Å)	*T* (°C)
DMPC	1.3 ${}^{a}$	36 ${}^{h}$	20
	2 ${}^{b}$	36 ${}^{h}$	23
	350 ${}^{c}$	36.7 ${}^{i}$	37
DPPC	9.2 ${}^{a}$	44 ${}^{j}$	37
	8 ${}^{b}$	44 ${}^{j}$	41
	46–178 ${}^{d}$	44 ${}^{j}$	40
TEMPO-DPPC	422 ${}^{a}$	44 ${}^{j}$	37
POPC	60,000 ${}^{c}$	39.1 ${}^{i}$	37
DSPC	312 ${}^{a}$	50 ${}^{k}$	41.5
Chol	200 ${}^{e}$	39.1 ${}^{i}$	50
	2 ${}^{f}$	38.6 ${}^{i}$	37
	1 ${}^{g}$	44 ${}^{l}$	37

DMPC, 1,2-dimyristoyl-sn-glycero-3-phosphocholine; DSPC,1,2-distearoyl-sn-glycero-3-phosphocholine. ${}^{a}$ Chemical label-free supported bilayer [71]; ${}^{b}$ free-floating lipid vesicles with a chemical label (NBD, 7-nitrobenz-2-oxa-1,3-diazol-4-yl) [72]; ${}^{c}$ free-floating lipid vesicles without a chemical label [73]; ${}^{d}$ free-floating lipid vesicles with a chemical label (TEMPO, *n*,*n*-dimethyl-n-(2’,2’,6’,6’-tetramethyl-4’-piperidyl)) [74]; ${}^{e}$ free-floating lipid vesicles without a chemical label [75]; ${}^{f}$ free-floating vesicles with cyclodextrin exchange [76]; ${}^{g}$ free-floating vesicles [77]; ${}^{h}$ [78]; ${}^{i}$ [34]; ${}^{j}$ [79]; ${}^{k}$ [80]; ${}^{l}$ [81].

Despite the discrepancies in absolute half-lives between different studies, there are common trends that are present among single studies; most notably, the dependence of flip/flop rate on bilayer thickness [71,72]. Phospholipids with longer acyl chains or, more specifically, bilayers with thicker hydrocarbon regions, show reduced flip/flop. This can be rationalized by the larger energetic cost of bringing the polar lipid headgroup through a longer hydrophobic path in a thicker membrane. Another contributing factor to flip/flop is the perturbation of bilayer integrity by the acyl chains, which need to be inverted during this process [73]. This may be facilitated by detergents, which have been postulated to promote transbilayer lipid movement by inducing transient hydrophobic structural defects in the membrane barrier [82].

**Figure 6:**
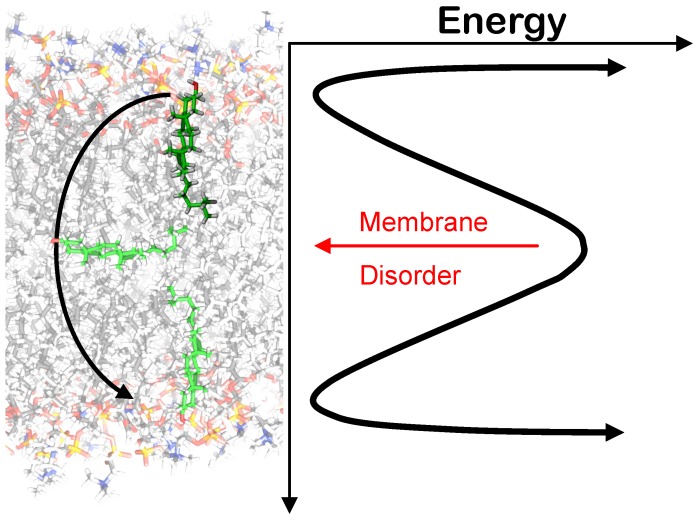
Energy profile for Chol flip/flop. The red arrow indicates that the barrier for flip/flop decreases as membrane disorder increases, as reported in [83,84].

The flip/flop rates of Chol have been determined in a number of different experimental methods and by MD simulations, which have been proven very valuable due to the complexity of probe-free studies. MD studies performed by Jo *et al.* and Bennet *et al.* yielded consistent average single molecule flipping for Chol flip/flop ranging from 1.4 ms–80 ns depending on temperature and membrane composition [83,84]. The prevailing trend in these studies is that Chol flip/flop increases with membrane disorder, which decreases with temperature and unsaturated lipid content (Figure 6).

Experimentally-determined Chol flip/flop rates are higher than predicted by MD. A study, from 1981, using Chol oxidase to monitor flip/flop put an upper limit of 1 min on the half-time [77]. This value is consistent with more recently-determined values by Leventis and Silvius, who put the halftime of flip/flop at 1–2 min using ${}^{3}$H-labeled Chol partitioning studies [76]. Strikingly, a recent neutron scattering study of Chol flip/flop has yielded a halftime of 200 min [75], highlighting the need for further study.

### 3.4. Membrane Proteins in Asymmetric Membranes

**Figure 7:**
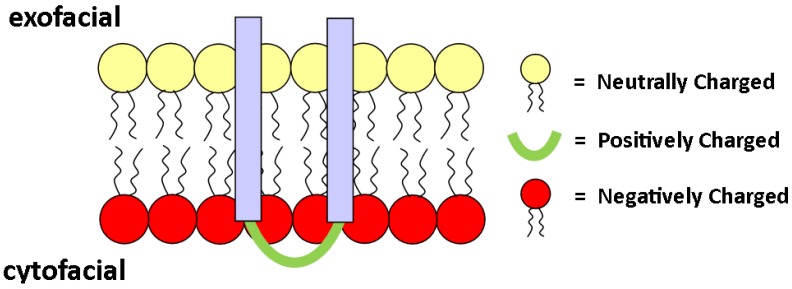
Schematic of an integral protein orienting itself according to electrostatic attraction between positive domains on the protein and negatively-charged lipids on the inner leaflet.

It is known that there is an asymmetric distribution of lipid charge across the bilayer, with a higher anionic charge, typically due to PS, at the cytoplasm-facing monolayer. The orientation of transmembrane (TM) proteins is often dictated by the location of charged lipid species. For example, many TM proteins carry a positive charge on their cytosolic domain, which likely helps the protein orient toward the inner leaflet due to the large PS content (negative charge density) of the cytosolic leaflet [85,86]; see Figure 7. Heijne and Gavel postulate that a positive inside rule for integral proteins is a universal property, and studies have shown that, in fact, statistically, a positive inside rule applies to eukaryotic cells [86,87]. However, the positive inside rule correlates stronger for prokaryotic cells [87].

In model lipid bilayers, there currently exist problems studying the inside-out rule, for integral proteins, due to a lack of control on lipid distribution in the systems. To the best of our knowledge, only one study has examined the reconstitution of protein in an asymmetric vesicle. London studied perfringolysin O (PFO) in symmetric and asymmetric vesicles composed of POPC/POPE/POPS/Chol(POPC, 1-palmitoyl-2-oleoyl-sn-glycero-3-phosphocholine; POPE,1-palmitoyl-2-oleoyl-sn-glycero-3-phosphoethanolamine; POPS, 1-palmitoyl-2-oleoyl-sn-glycero-3-phospho-L-serine) , where POPC was located on the outer leaflet and POPE:POPS was on the inner leaflet [85]. The study revealed the conformational behavior of PFO differed for asymmetric and symmetric vesicles, as well as symmetric vesicles composed of either inner or outer leaflet lipids [85]. The aforesaid results demonstrate, specifically, that pore formation in asymmetric vesicles follows a different mechanism than in symmetric ones. More broadly, these results show the importance of new membrane platforms for the study and understanding of integral proteins.

## 4. Final Thoughts

First and foremost, asymmetric bilayers are a much better biological mimetic than the symmetric bilayer counterpart. It is presently unclear how much insight has been accomplished over the decades of biophysical research into symmetric membrane mimetics that can be transferred to asymmetric systems. It almost appears as if research has taken a leap into the dark, which is scientifically quite exciting. In any case, the ability to construct asymmetric vesicles is an important step forward, toward a better understanding of the structure and function of cell membranes, since, with few exceptions, biological membranes are strongly asymmetric.

While the reasons for this asymmetry remain shrouded in mystery, much can be learned about the fundamental physical and chemical properties of asymmetric bilayers, as reviewed above. Further studies in this direction are needed and will be spurred as generating asymmetric vesicles becomes more robust and analyzing asymmetry develops further. Concise answers will be sought and found for the nature and mechanisms of interleaflet coupling and whether or not phase separation in one leaflet induces demixing of lipid components in the other leaflet. Lipid flip/flop rates, which have historically been challenging to obtain, will be quantified, allowing researchers to address long-standing questions regarding the influence of bilayer structure and composition (including the presence of transmembrane proteins). Eventually, this will aid our understanding of physiological processes on the cellular level, including, e.g., raft-formation and transmembrane signaling, and will assist the design of novel drugs that will specifically interfere with membrane function in the case of disease [88].
